# Buffalo Academy of Medicine

**Published:** 1906-02

**Authors:** Franklin W. Barrows


### Buffalo Academy of Medicine.

Reported by FRANKLIN W. BARROWS, M. D., Secretary protem.

A SPECIAL meeting of the Academy was held at the Public Library Building December 28, 1905, which was called to order at 9.05 p. m., by the president, Dr. H. U. Williams.

The first paper of the evening was presented by Dr. William P. Spratling, Medical Superintendent of the Craig Colony for Epileptics, Sonyea, N. Y., and was entitled

### REMARKS ON EPILEPSY

Abstract:—The care of epileptics in colonies was taken up in Germany in 1867. Ohio began the work in 1890 and Craig Colony was opened in 1894. Today five states have colonies. There are many different epilepsies. Our present classification is based on symptomatology; the etiology has yet to be studied. Heredity is the chief factor in 16 per cent. of cases, alcoholism in 15 per cent, tuberculosis, rheumatism and nervous diseases in 55 to 60 per cent, and next in importance to these factors are birth injuries, dentition and specific fevers.

The great epileptic epoch is the time of puberty, from the ages of 12 to 16. Up to the 20th year the incidence of epilepsy is about equal for both sexes; after this year heredity shows very little influence. In 83 per cent of cases studied, the disease came on before the 20th year. After this time, for every 100 men there were 80 women seized with the disease. Alcohol, syphilis and head injuries are the chief causes at this epoch; women are subject during the puerperium. The epilepsy of senility is of all kinds most amenable to treatment, and is often palliated.

The lesion of epilepsy is in the brain cortex. Pathology has taught us much more about the results of this disease than about the cause. We must study the clinical features and physiological chemistry of our case,—the living instead of the dead. We shall in no other way discover the toxins and other predisposing causes.

Bromides are much abused in treating epilepsy. The best treatment is by diet, starvation, baths and other means of elimination. About 60 per cent of cases have an aura, occurring from one second to several months before the fit. The visual aura is commonest. Some patients have auras of all the senses in rotation. There are many varieties of cephalic, gastric and motor auras.

Prognosis. Epilepsy is in the category of curable diseases. We say that about five to ten per cent can be cured. Less than one-half of the cases received at Craig Colony are in a stage where cure is possible; of these we have discharged about five and one-half per cent. as cured. It takes time, often years. If I had predicted, 25 years ago, that we would today be curing 20 to 25 per cent of our tuberculosis cases you would not have believed me; according to statistics of Dr. Trudeau, that is what we are doing now. When we treat epilepsy as broadly and rationally as we do tuberculosis, by planning out a course of right living, we will cure as large a percentage as we do now of insanity cases. Surgical treatment offers a great field. Brilliant work will sometime be done in the cases of infants and children with blood clots on the brain; some other idiopathic cases are also amenable to operation. The keynote of our work today is prevention.

Dr. Spratling next introduced Dr. Walter G. Chase, of Boston, and spoke in the highest terms of his excellent work in securing living pictures of epileptic convulsions at Craig Colony.

Dr. Chase made a short address on "The use of the biograph in medicine", in which he showed how to utilize this instrument in class instruction, in demonstrating various gaits and other motions, in illustrating obstetrical technic, artificial respiration, physiological demonstrations, and the like.

Dr. Chase closed the program with an exhibition of biograph pictures showing a large number and variety of epileptic convulsions, in addition to which he showed various varieties of gait, athetosis, chorea, rhythmic movements of insanity, nystagmus, etc. The pictures were most excellent from an artistic point of view and afforded a rare and unique opportunity for the study of the various motions depicted. While the pictures were on the screen Dr. Spratling gave a running comment, describing the cases and demonstrating the characteristic features of each. This innovation in the work of the Academy was greatly appreciated and elicited generous applause and many questions.

On motion of Dr. Roswell Park, seconded by Dr. Charles Cary, the thanks of the meeting were unanimously extended to the speakers.

The Academy adjourned at 11:10 p. m. Attendance 65.

